# Knockdown of long non-coding RNA LEF1-AS1 attenuates apoptosis and inflammatory injury of microglia cells following spinal cord injury

**DOI:** 10.1186/s13018-020-02041-6

**Published:** 2021-01-06

**Authors:** Sheng-Yu Cui, Wei Zhang, Zhi-Ming Cui, Hong Yi, Da-Wei Xu, Wei Liu, Xin-Hui Zhu

**Affiliations:** Department of Orthopedic, Nantong First People’s Hospital & The Second Affiliated Hospital of Nantong University, No. 6 Haierxiangbei Road, Nantong, 226001 Jiangsu Province China

**Keywords:** Spinal cord injury, LEF1-AS1, miR-222-5p, ceRNA, Inflammation, Apoptosis

## Abstract

**Background:**

Spinal cord injury (SCI) is associated with health burden both at personal and societal levels. Recent assessments on the role of lncRNAs in SCI regulation have matured. Therefore, to comprehensively explore the function of lncRNA LEF1-AS1 in SCI, there is an urgent need to understand its occurrence and development.

**Methods:**

Using in vitro experiments, we used lipopolysaccharide (LPS) to treat and establish the SCI model primarily on microglial cells. Gain- and loss of function assays of LEF1-AS1 and miR-222-5p were conducted. Cell viability and apoptosis of microglial cells were assessed via CCK8 assay and flow cytometry, respectively. Adult Sprague-Dawley (SD) rats were randomly divided into four groups: Control, SCI, sh-NC, and sh-LEF-AS1 groups. ELISA test was used to determine the expression of TNF-α and IL-6, whereas the protein level of apoptotic-related markers (Bcl-2, Bax, and cleaved caspase-3) was assessed using Western blot technique.

**Results:**

We revealed that LncRNA LEF1-AS1 was distinctly upregulated, whereas miR-222-5p was significantly downregulated in LPS-treated SCI and microglial cells. However, LEF1-AS1 knockdown enhanced cell viability, inhibited apoptosis, as well as inflammation of LPS-mediated microglial cells. On the contrary, miR-222-5p upregulation decreased cell viability, promoted apoptosis, and inflammation of microglial cells. Mechanistically, LEF1-AS1 served as a competitive endogenous RNA (ceRNA) by sponging miR-222-5p, targeting RAMP3. RAMP3 overexpression attenuated LEF1-AS1-mediated protective effects on LPS-mediated microglial cells from apoptosis and inflammation.

**Conclusion:**

In summary, these findings ascertain that knockdown of LEF1-AS1 impedes SCI progression via the miR-222-5p/RAMP3 axis.

## Introduction

Spinal cord injury (SCI) is, in most cases, caused by trauma and is associated with health burden both at personal and societal levels [1, 2]. Studies have shown that over 10,000 cases of SCI are annually reported in the USA, with an increasing trend [3]. SCI is characterized by a primary mechanical insult to the spinal cord and subsequently causes secondary injury. Secondary injury may instigate apoptotic and necrotic cell death and excessive inflammatory response [4].

During a secondary injury, inflammatory cytokines induce microglial cells, and apoptosis is mainly used in the pathogenesis of SCI [5]. Therefore, additional research on the pathogenesis of SCI is critically important in discovering effective methods to prevent and manage of SCI.

Present studies have identified long non-coding RNAs (lncRNAs, > 200 nt), a class of non-coding RNAs, to contribute to inflammatory injury [6, 7]. Numerous reports have been published on the function of lncRNAs in SCI [8–10]. For instance, Zhou et al. [8] suggested that lncGBP9 could derives macrophages toward a phenotype conducive for spinal cord injury repair via miR-34a/STAT1/STAT6 and SOCS3 axis. Xia et al. [9] revealed that lncRNA CCAT1 protects astrocytes against OGD/R-induced damage by targeting the miR-218/NFAT5-signaling axis. Zhao et al. [10] in their study, revealed that lncRNA XIST was significantly increased in SCI, whereas knockdown of XIST potentially delayed SCI progression.

Lymphoid enhancer-binding factor 1 (LEF1) antisense RNA 1 (LEF1-AS1) is a newly discovered lncRNA [11]. In recent years, researches have revealed that lncRNA LEF1-AS1 may play a vital role in the progression of multiple tumors [12–14]. However, the role of lncRNA LEF1-AS1 in SCI, especially in regulating microglia cells inflammation and apoptosis after SCI, is elusive.

MicroRNAs (miRNAs) are single-stranded non-coding small-molecule RNA with 18 to 24 base pairs in length, thus are mostly localized in the intergenic region [15, 16]. MiRNAs regulate gene expression by inhibiting mRNA translation or cleaving messenger RNA (mRNA) at the post-transcription level, therefore, facilitates cell growth and differentiation, stem cell differentiation, cell invasion, and apoptosis [17]. Moreover, increasing evidence suggests that miRNAs are inextricably linked to SCI occurrence. For example, An et al. [18] found that miR-466c-3p protects neuronal apoptosis and improves functional recovery of post spinal cord injury. A study by Wu et al. [19] reported that miR-615 could regulate neural stem cell differentiation by targeting LRR and Ig domain-containing NOGO receptor-interacting protein 1 (LINGO-1). As a member of miRNAs, miR-222-5p exerts an anti-apoptotic activity in non-small cell lung cancer [20] and B cell lymphoma [21]. However, the role of miR-222-5p in SCI progression remains elusive.

The purpose of this study was to explore the role of lncRNA LEF1-AS1 and miR-222-5p in SCI.

## Materials and methods

### In vitro study

#### Primary microglial cells isolation and culture

Embryonic Wistar rats were sourced from Beijing Vital Laboratory Animal Technology Company (Beijing, China). The central nervous system of rats was stripped off, digested in 0.025% trypsin (60 min, 37 °C), and centrifuged at 1800×*g* for 5 min before cell collection. After that, collected cells were maintained at 37°C with 5% CO_2_ in high-glucose Dulbecco’s modified Eagle’s medium (DMEM, Gibco, Grand Island, NY, USA) containing 20% fetal bovine serum (FBS). Procedurally, the culture medium was replenished every 3 days, and the cells of passage 3–5 (P3-P5) were used in subsequent experiments [10].

#### Cell transfection and treatments

Short-hairpin RNA (shRNA) targeting LEF1-AS1 (sh-LEF1-AS1), siRNA for LEF-AS1, pcDNA-RAMP3, and their negative controls (vector and sh-NC) were obtained from GeneCopoecia (Guangzhou, China). miR-222-5p mimics and inhibitors, and their negative controls were obtained from Genepharma (Shanghai, China). Experimentally, microglial cells seeded in 24-well plates were transfected with the above expressing vectors using Lipofectamine 3000 (Invitrogen, CA, USA). A fresh complete culture medium was introduced after 48 h and maintained for another 24 h. RT-PCR was used to verify the transfection efficiency. Chondrocytes were treated with 100 ng/mL of lipopolysaccharide (LPS, Sigma-Aldrich, Darmstadt, Germany, 1 μg/ml) for 24 h to induce in vitro SCI model.

#### Cell count kit-8 (CCK-8) assay

Here, equal amounts of cells (5 × 10^3^ cells/well) were seeded in 96-well plates. Normal and transfected cells were treated with 1 μg/ml of LPS for 24 h, then cell proliferation was examined using CCK8 kit (CCK-8; Dojindo Molecular Technologies, Gaithersburg, USA) at 37°C with 5% CO_2_. Microplate reader (Tecan Infinite M200 Micro Plate Reader; LabX, Switzerland) was used to measure absorbance value at 450 nm of each well. Resultant measurements were assessed in triplicate and experiments repeated thrice.

#### Apoptosis experiment

Firstly, apoptosis was demonstrated by the apoptosis kit (FITC Annexin V Apoptosis Detection Kit I, Boster, Wuhan, China) following the manufacturer’s instructions. Herein, cells in the logarithmic growth phase were trypsinized and inoculated into 6-well plates at 1 × 105 per well, following their culturing at 37 °C for 24 h. Afterward, the transfected treated cells were collected and washed with PBS. Subsequently, the transfected microglial cells were suspended in annexin-binding buffer and stained with 5 μL of FITC Annexin V and 0.1 μg of PI in the dark for 15 min at room temperature. We diluted the stained apoptotic cells in PBS and were detected using flow cytometry (BD Biosciences, San Jose, CA).

#### RT-qPCR

Total RNA in grouped and treated cells were extracted using TRizol reagent and RNeasy® Plus Micro Kit (QIAGEN, Hilden, Germany) respectively. Total RNAs were reverse-transcribed using the cDNA Reverse Transcription Kit (Takara, Japan) as per the manufacture's protocol. Real-time quantitative PCR was performed using SYBR Green real-time PCR kit (TaKaRa, Dalian, China) with U6 and GAPDH as internal references. LightCycler 480 real-time PCR instrument was used for analysis with a 2^−ΔΔCt^ computation method. The experimental study was conducted in triplicate. Primers for each gene are shown in Table 1.

**Table 1:** The primers for each gene

Gene	Sequence
GAPDH	forward: 5′-GAAGATGGTGATGGGATTTC-3′
	reverse: 5′-GAAGGTGAAGGTCGGAGT-3′
U6	forward: 5′-CTCGCTTCGGCAGCACA-3′
	reverse: 5′-AACGCTTCACGAATTTGCGT-3′
LEF-AS1	forward: 3′-TGT GAC TCC AGA GGC GGA AC-5′
	reverse: 3′-AGG AGG CGG CAA GAA GAA GG-5′
miR-222-5p	forward: 5′-TGGCCTCGAGATGTGCTTCAG-3′
	reverse: 5′-TCTCCTTGGCGGCCGCACTTCCTTC-3
RAMP3	forward: 5′-ACACCCGTGAGAGAGACTTG-3′
	reverse: 5′-AAGTCAGTCGGGAAGGAAGG-3′

#### Western blot

RIPA lysis buffer (R0020, Solarbio, Beijing, China) with fresh protease inhibitor with 0.1% phenylmethanesulfonyl fluoride (PMSF, Solarbio) was used to separate total protein. Afterward, 50 μg of total protein was loaded to a 12% polyacrylamide gel and electrophoresed at 100 V for 2 h, then transferred to polyvinylidene fluoride (PVDF) membranes. Thereafter, PVDF membranes were incubated with primary antibodies of anti-cleaved caspase 3 (Abcam, concentration 1: 1000), anti-Bcl-2 (Abcam, concentration 1: 1000), anti-Bax (Abcam, concentration 1: 1000), and anti-GAPDH (Abcam, concentration 1: 1000) at 4 °C overnight. Subsequently, PVDF membranes were washed thrice (15 min/wash) with TBST and incubated with horseradish peroxidase (HRP)-labeled anti-rabbit secondary antibody (Proteintech, Wuhan, China, concentration 1: 3000) at room temperature for 1 h. Finally, Western blot visualization was performed using ECL reaction (Amersham ECL Prime Western Blotting Detection Reagent) and gray values of the protein expressions quantified by Image-Pro Plus 6.0 software (NIH Image, USA).

#### Dual-luciferase reporter assay

Wild-type (WT) or mutant (MUT) LEF-AS1/RAMP3 was cloned to Sac I-Xba I site of pmirGLO Vector (Promega, Madison, WI) to generate reporter plasmids. Afterward, WT or MUT reporter vectors were co-transfected with miR-222-5p mimics into primary microglial cells according to their binding sites. Dual-luciferase reporter assay (Promega, Madison, WI, USA) was conducted to test Firefly and Renilla luciferase activities 48 h post-transfection. Luciferase activity was calculated as the ratio of firefly luciferase intensity to renilla luciferase intensity.

#### RIP and RNA pull-down experiment

Magna RIP RNA-Binding Protein Immunoprecipitation Kit (Millipore, MA, USA) was used to perform the RIP process, 48 h post-cell transfection. Microglial cells incubated with anti-Ago2 antibody (Millipore) or negative control IgG (Millipore) and RIP enrichment were calculated as LEF-AS1 or RAMP3 relative to the input control.

Subsequently, miR-222-5p-WT and miR-222-5p-MUT were in vitro transcribed, respectively. Transcribed RNA was labeled with biotin using Biotin RNA Labeling Mix (Roche, AG, Basel, Switzerland) and T7 RNA polymerase (Roche) before purification. The Probes incubated at room temperature for 2 h with streptavidin-coated magnetic beads (Sigma) were later incubated with RASFs lysates. After washing with PBS, RNA complexes bound to the beads were eluted and extracted to determine RAMP3 expression with RT-qPCR. Beads without probe incubation were treated as a control group.

### In vivo study

#### Specimen collection

Experimental studies were approved by the Ethics Committee of the Nantong First People’s Hospital (NFPH-2019-0034). We procured 40 male Sprague–Dawley (SD) rats (280–300 g) from Beijing Vital Laboratory Animal Technology Co. Study rats were housed under conditions following guidelines on Care and Use of Laboratory Animals issued by the Chinese Council on Animal Research and Guidelines on Animal Care. The SCI model was induced as previously described [10]. Rats were anesthetized with 10% chloral hydrate and placed in a prone position. Thereafter, the vertebral column was exposed between T9 and T10, whereby a total laminectomy performed at the T10 level. SCI was induced by New York University Impactor device (NYU, New York, NY, USA) using a 10-g-rod at a 25-mm-drop height. Sham group received total laminectomy without hitting the T10 level. Additionally, at 3 days post-operation, we assessed hind-limb locomotor function in all rats using Basso, Beattie, Bresnahan Locomotor Rating Scale (BBB).

### Statistical analysis

Differences between groups were analyzed using SPSS 21.0 software (SPSS, IBM, Armonk, NY, USA). Unpaired Student’s *t* test and one-way analysis of variance (ANOVA) followed by Bonferroni’s post hoc analysis were used to compare two or multiple groups, respectively. Independently experiments conducted in triplicates were analyzed. *P* < 0.05 was considered statistically significant.

## Results

### LEF1-AS1 upregulated in SCI models

**Fig. 1:**
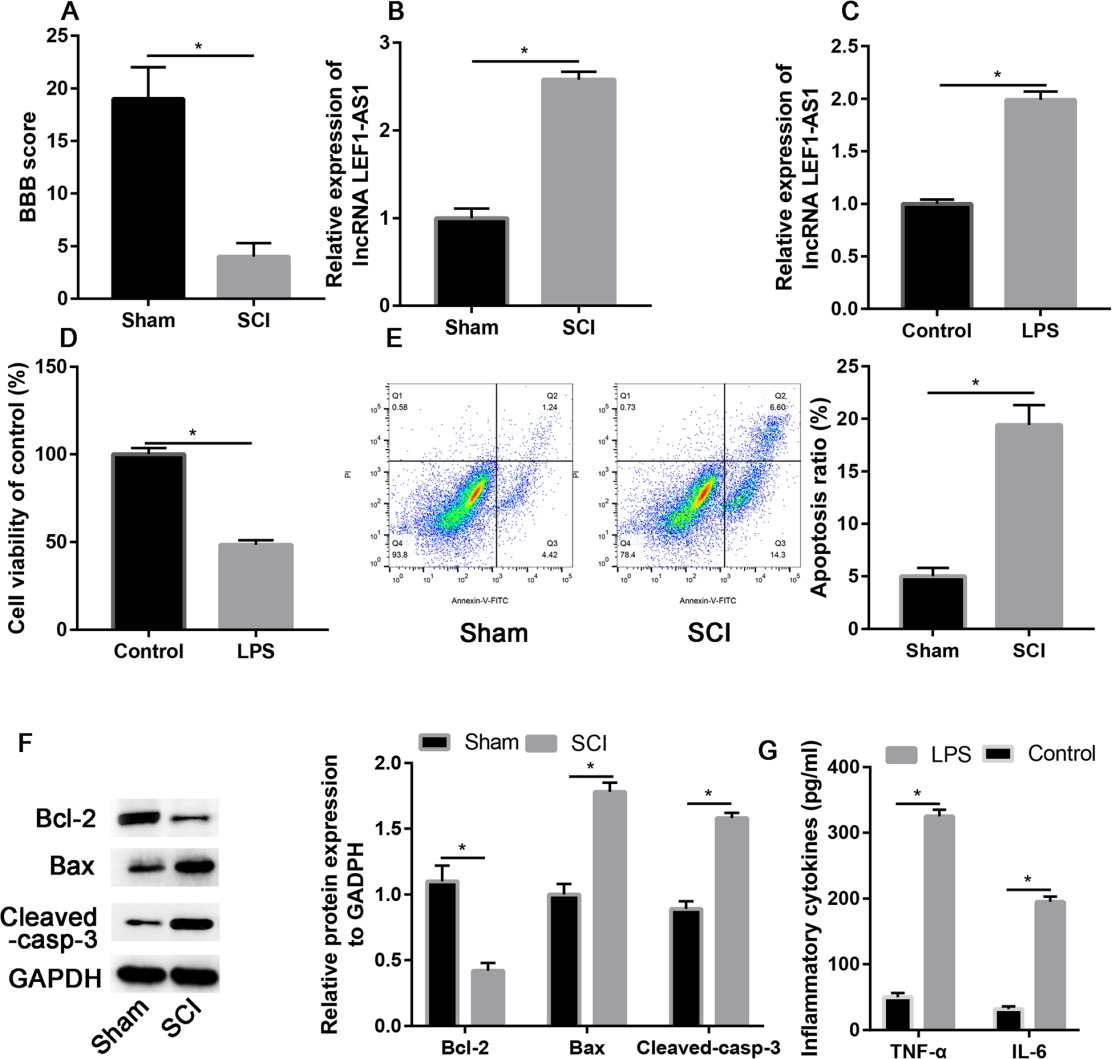
LncRNA LEF-AS1 was upregulated in the SCI model in vivo and in vitro. **a** BBB scores were measured in sham and SCI groups. **b** Relative expression of lncRNA LEF-AS2 in sham and SCI groups. **c** Relative expression of lncRNA LEF-AS2 in control and LPS-treated microglia cells. **d** Cell viability in control and LPS-treated microglia cells measured by CCK-8 assay. **e** Apoptosis ratio in control and LPS-treated microglia cells measured by Annexin V/FITC apoptosis assay. **f** Western blot assay to measure the protein expression of apoptotic markers (Bax, Bcl-2, and cleaved-caspase-3). **g** Inflammatory cytokines (TNF-α and IL-6) expression levels in control and LPS-treated microglia cells (*P* < 0.05)

The well-established model of SCI was used to investigate the expression of lncRNA LEF1-AS1 in SCI mode. Our study findings showed a highly significant three-fold reduction of BBB scores in the SCI group than in the sham group (*P <* 0.001, Fig. 1a), indicating a successfully established SCI animal model. The expression of a lncRNA LEF1-AS1 was significantly higher in the SCI animal model than in the sham group (*P* = 0.002, Fig. 1b). Meanwhile, the result showed that lncRNA LEF1-AS1 was significantly increased in LPS-treated group (*P* = 0.021, Fig. 1c). Microglial cell viability decreased by 48.4% from the control value after cotreatment with LPS (*P* = 0.006, Fig. 1d). Flow cytometry analysis demonstrated an increased fraction of apoptotic cells in LPS-treated microglial cells (Fig. 1e). Cleaved caspase 3 and Bax, markers of apoptosis, were significantly increased in the LPS-treated groups compared to the control group (*P* = 0.014 and *P* = 0.024, Fig. 1f). On the contrary, Bcl-2, an anti-apoptotic marker, decreased in LPS-treated groups compared to the control group (*P* < 0.001, Fig. 1f). Moreover, TNF-α and IL-6 were significantly increased post-treatment with LPS (*P* = 0.013 and *P* = 0.005, Fig. 1g). Cumulatively, these findings indicated that lncRNA LEF1-AS1 expression was significantly upregulated in the SCI tissues and LPS-induced microglial cells.

### Knockdown of LEF1-AS1 exert a protective role on LPS-induced apoptosis

**Fig. 2:**
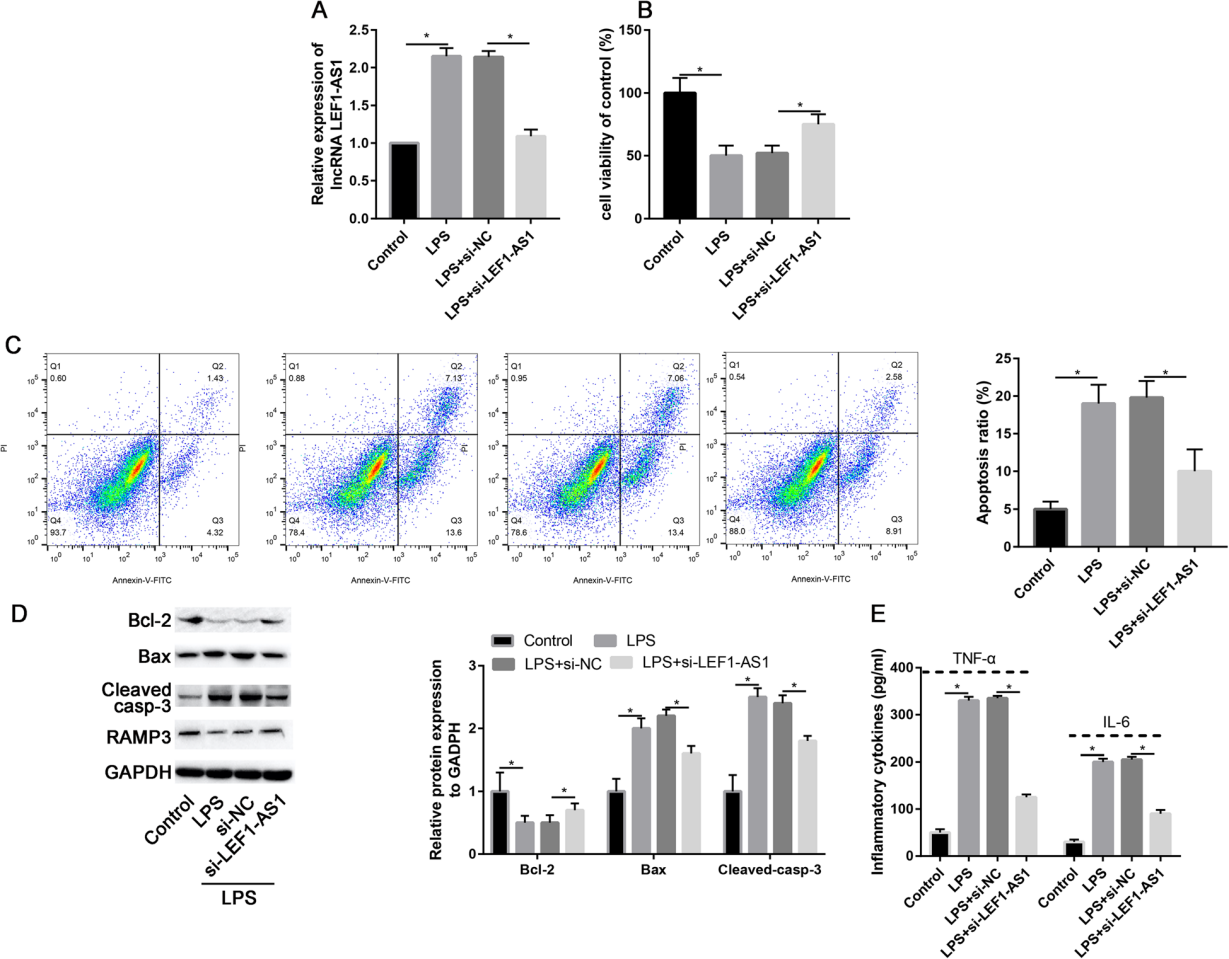
Knockdown of lncRNA LEF-AS1 could decrease the LPS-induced microglia cells apoptosis. **a** The expression level of lncRNA LEF-AS1 in si-NC and si-LEF-AS1 groups. **b** Cell viability in LPS and/or transfected with si-LEF-AS1. **c** Apoptosis ratio in LPS and/or transfected with si-LEF-AS1 measured by Annexin V/FITC apoptosis assay. **d** Western blot assay measuring protein expression of apoptotic markers (Bax, Bcl-2, and cleaved-caspase-3) in microglia cells transfected with si-LEF-AS1. **e** Inflammatory cytokines (TNF-α and IL-6) expression levels in microglia cells transfected with si-LEF-AS1. *P < 0.05

Further investigations on functions and mechanisms of lncRNAs LEF1-AS1 in LPS-treated microglial cells inflammatory injury and apoptosis were assessed through the construction of siRNA to knockdown lncRNA LEF1-AS1. Knockdown efficacy was evaluated in transfected cells by quantitative PCR analysis (Fig. 2a). Compared with LPS+si-NC, LPS+si-LEF-AS1 could decrease the lncRNA LEF-AS1 (*P* = 0.017).

On the other hand, CCK-8 assay was used to measure cell viability. Consequently, significant downregulation of cell viability was increased in the LPS treated cells; hence, knockdown of LEF1-AS1 by si-LEF1-AS1 could partially reverse LPS inhibitory effects on microglial cells viability (*P* < 0.001, Fig. 2b). Annexin-V/FITC and PI staining analysis were used as confirmatory tests on the effect of lncRNA LEF1-AS1 on apoptosis. Results indicated that LPS significantly increased apoptosis rate as compared to the control group (*P* = 0.005, Fig. 2c), co-transfection with si-LEF1-AS1 could partially inhibit LPS-mediated apoptosis (*P* < 0.001, Fig. 2c). Also, western blot results showed that LPS could cause an increase in Bax and cleaved caspase-3 proteins expression, thus, significantly inhibit protein expression of Bcl-2 induced by LPS (*P* = 0.002 and *P* = 0.001, Fig. 2d).

Compared with LPS group, transfection with si-LEF1-AS1 could partially reduce LPS-induced overexpression of Bax and cleaved caspase-3 protein expression (*P* = 0.015 and *P* = 0.007, Fig. 2d). Also, si-LEF1-AS1 could partially increase the downregulated Bcl-2 level caused by LPS (*P* = 0.012, Fig. 2d). Consistently, the up-regulation of TNF-α and IL-6 induced by LPS were partially attenuated by si-LEF1-AS1 (*P* = 0.017 and *P* = 0.037, Fig. 2e). Taken together, results indicated that, knockdown of LEF1-AS1 could protect LPS-induced microglial cells apoptosis and inflammatory injury.

### LncRNA LEF1-AS1 directly target with miR-222-5p

**Fig. 3:**
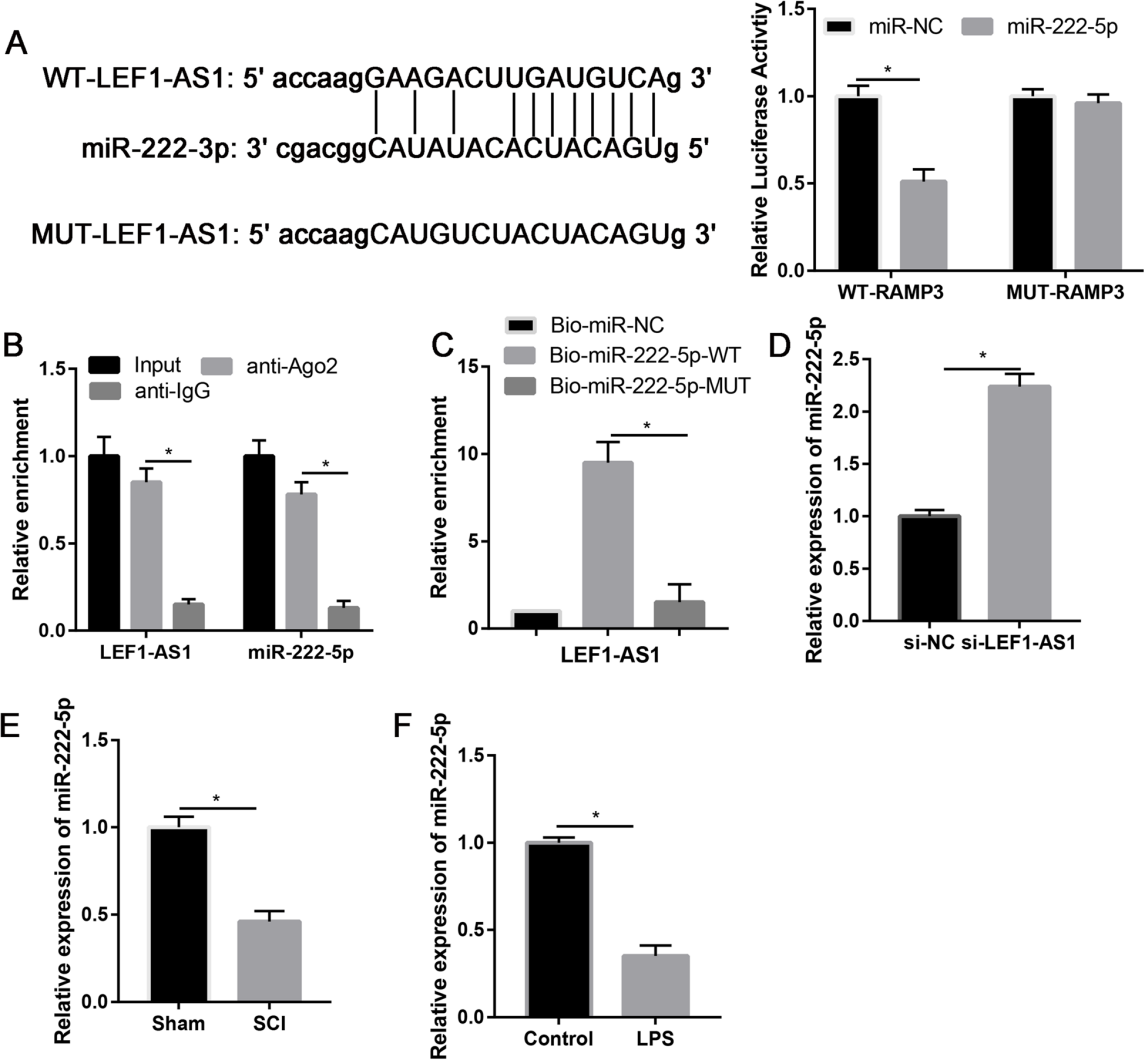
lncRNA LEF-AS1 directly targets miR-222-5p. **a** Starbase (http://starbase.sysu.edu.cn/) revealed that LEF-AS1 contains binding sequences with miR-222-5p. **b** RNA immunoprecipitation (RIP) assay to assess enrichment of LEF-AS1 and miR-222-5p in microglial cells. **c** RNA pull-down performed to assess enrichment of LEF-AS1 in microglial cells. **d** Relative expression of miR-222-5p in si-NC and si-LEF-AS1 groups. **e** Relative expression of miR-222-5p in sham and SCI groups. **f** Relative expression of miR-222-5p in control and LPS-treated microglia cells; *P < 0.05

We found that there were 11 potential binding sites of miR-222-5p in LEF1-AS1 (Fig. 3a). Luciferase activity assay revealed that miR-222-5p suppressed LEF1-AS1 3′-UTR luciferase activity (*P* = 0.029), while it was not effective on MUT LEF1-AS1 3′-UTR luciferase activity as compared to control in microglial cells (*P* = 0.108, Fig. 3a). RIP assay (Fig. 3b) and RNA pull-down analysis (Fig. 3c) determined that miR-222-5p mimic could significantly increase the LEF1-AS1 expression (*P* = 0.024). Moreover, the expression of miR-222-5p was significantly increased after knockdown of LEF1-AS1 than si-NC (*P* = 0.021, Fig. 3d). Both in vivo and in vitro models on the expression of miR-222-5p in SCI were researched. However, results indicated that miR-222-5p was significantly downregulated in the SCI model (*P* = 0.018, Fig. 3e) and LPS-treated microglial cells (*P* = 0.026, Fig. 3f). According to the cumulative data, lncRNA LEF1-AS1 directly targets with miR-222-5p in microglial cells to regulate microglial cells’ apoptosis.

### miR-222-5p directly target with RAMP3

**Fig. 4:**
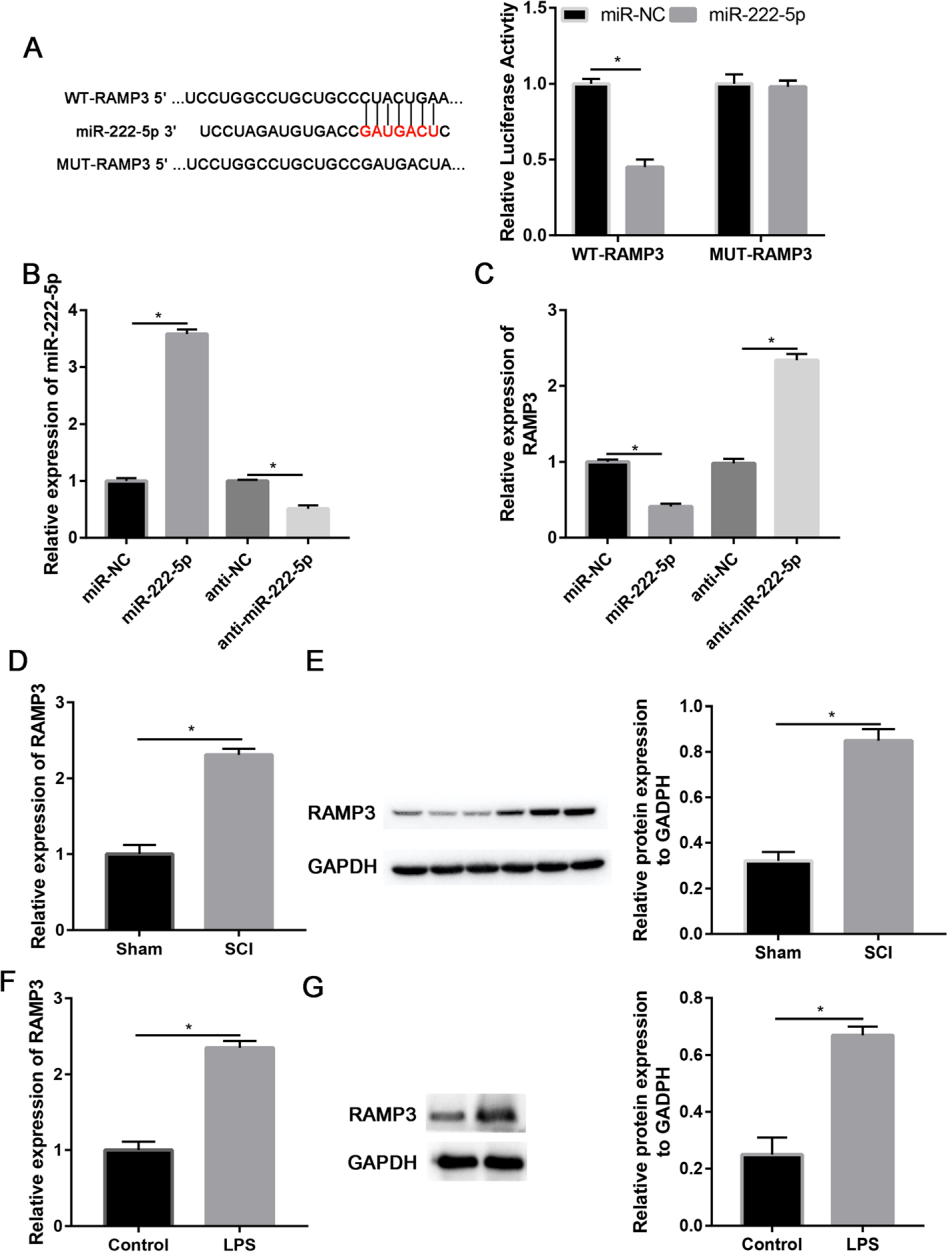
miR-222-5p directly targets RAMP3. **a** Starbase (http://starbase.sysu.edu.cn/) showing RAMP3 containing binding sequences with miR-222-5p. **b** miR-222-5p expression in miR-222-5p mimic and anti-miR-222-5p groups. **c** Relative RAMP3 expression in miR-222-5p mimic and anti-miR-222-5p groups. **d** Relative mRNA expression of RAMP3 in sham and SCI groups. **e** Relative protein expression of RAMP3 in sham and SCI groups. **f** Relative mRNA expression of RAMP3 in control and LPS-treated microglia cells. **g** Relative protein expression of RAMP3 in control and LPS-treated microglia cells; *P < 0.05

RAMP3 was predicted as a direct target of miR-222-5p through three bioinformatics tools (TargetScan, miRDB, and miRanda) (Fig. 4a). Luciferase activity assay revealed that miR-222-5p decreased MUT RAMP3 3′-UTR luciferase activity (*P* = 0.004), while it was not effective on MUT RAMP3 3′-UTR luciferase activity in microglial cells (*P* = 0.157, Fig. 4a). Therefore, miR-222-5p mimic and inhibitor were constructed, respectively. miR-222-5p was highly increased than reduced after transfection of miR-222-5p mimic and inhibitor, respectively (*P* = 0.017, Fig. 4b). Furthermore, miR-222-5p mimic suppressed RAMP3 gene expression, whereas miR-222-5p inhibitor, enhanced RAMP3 gene expression (*P* = 0.029, Fig. 4c).

As indicated in Fig. 4d, e, RAMP3 mRNA and protein expression were upregulated in the spinal cord tissue of the SCI rat model compared to the sham group (*P* = 0.031). Also, RAMP3 mRNA and protein expression were upregulated in LPS-treated microglial cells than in the control group (Fig. 4f, g). In general, our data show that miR-222-5p directly target with RAMP3.

### LncRNA LEF1-AS1 act as a ceRNA by binding miR-222-5p and could repress RAMP3 expression

**Fig. 5:**
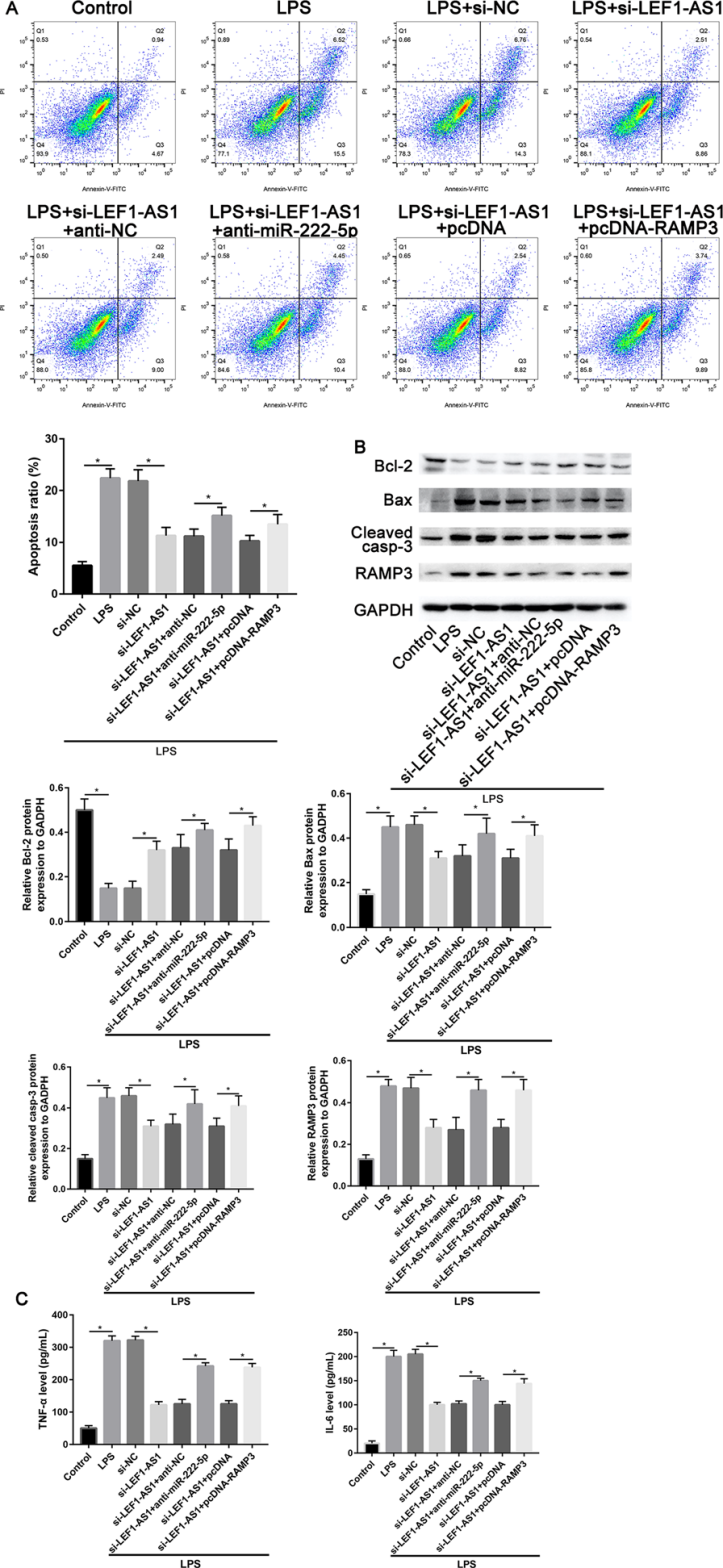
LncRNA LEF-AS1 acted as a ceRNA of miR-222-5p to regulate LPS-induced microglia cells apoptosis. **a** Apoptosis ratio in anti-miR-222-5p and pcDNA-RAMP3 transfected microglia cells. **b** Western blot assay to measure protein expression of apoptotic markers (Bax, Bcl-2, and cleaved-caspase-3) in microglia cells transfected with anti-miR-222-5p and pcDNA-RAMP3. **c** Inflammatory cytokines (TNF-α and IL-6) expression levels in microglia cells transfected with anti-miR-222-5p and pcDNA-RAMP3. *P < 0.05

According to Annexin-V/FITC and PI staining analysis, they confirmed that knockdown of miR-222-5p or RAMP3 overexpression could partially restore inhibitory apoptotic effects of si-LEF1-AS1 on LPS induced microglial cells (*P* < 0.001, Fig. 5a). Transfection si-LEF1-AS1 could downregulate the protein expression of Bax and cleaved caspase-3 when compared with the LPS group (*P* < 0.001). However, co-transfection with miR-222-5p inhibitor or RAMP3 overexpression plasmid could increase Bax and cleaved caspase-3 expression (*P* < 0.001, Fig. 5b).

Notably, transfection si-LEF1-AS1 could highly upregulate protein expression of Bcl-2 as compared with LPS-treated group. However, it is also determined that co-transfection with miR-222-5p inhibitor or RAMP3 overexpression plasmid could decrease the Bcl-2 expression (*P* = 0.029, Fig. 5b).

Consistently, the upregulation of TNF-α and IL-6 induced through LPS were partially attenuated by si-LEF1-AS1. Herein, the inhibitory effects of si-LEF1-AS1 were partially reversed by transfection with miR-222-5p inhibitor or RAMP3 overexpression plasmid (*P* = 0.017, Fig. 5b).

### LEF1-AS1 knockdown restore the function of SCI rats

**Fig. 6:**
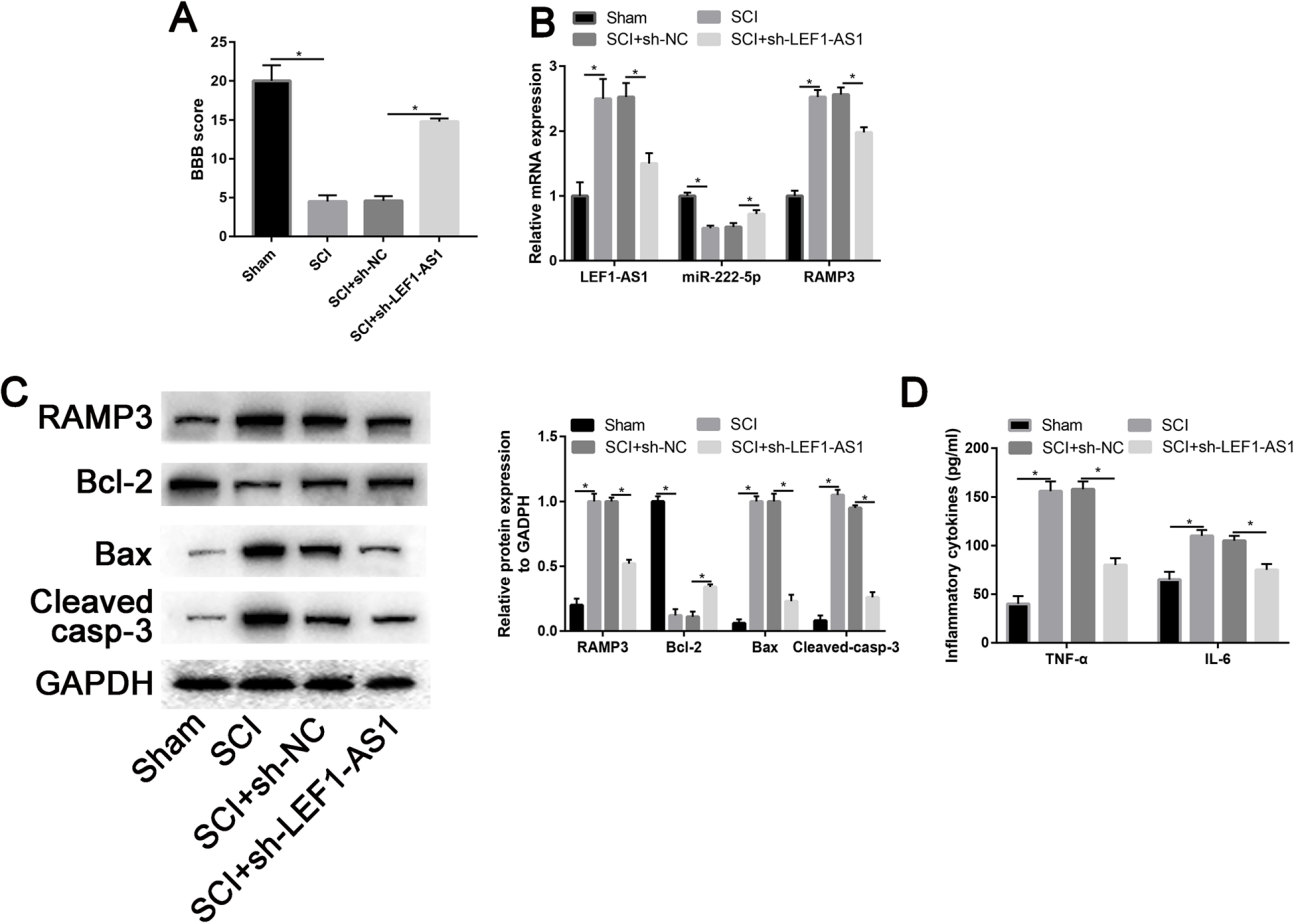
Knockdown of lncRNA LEF-AS1 restored functionally recovery in SCI rats. **a** BBB scores in SCI and transfected with si-LEF-AS1. **b** Relative expression of LEF-AS1, miR-222-5p and RAMP3 in SCI and transfected with si-LEF-AS1. **c** Western blot assay to measure the protein expression of apoptotic markers (Bax, Bcl-2, and cleaved-caspase-3) in SCI and transfected with si-LEF-AS1; d, inflammatory cytokines (TNF-α and IL-6) expression levels in SCI and transfected with si-LEF-AS1, *n* = 5, *P < 0.05

Our data revealed that administering sh-LEF1-AS1 could partially restore BBB scores in SCI rats (*P* = 0.007, Fig. 6a). Moreover, LEF1-AS1 overexpression plasmid could partially increase the expression of miR-222-5p while decreasing the expression of RAMP3 (*P* = 0.017, Fig. 6b). Western blot results determine that protein expression of Bax and cleaved caspase-3 were partially decreased whereas Bcl-2 was partially increased in the LEF1-AS1 knockdown group. Furthermore, TNF-α and IL-6 were partially decreased in the LEF1-AS1 knockdown group (*P* = 0.034, Fig. 6c).

## Discussion

Based on the findings, lncRNA LEF1-AS1 was upregulated in the SCI tissues, and knockdown of lncRNA LEF1-AS1 highly attenuated apoptosis of microglia cells. Furthermore, mechanistically, we demonstrated that lncRNA LEF1-AS1 functions as a competing endogenous RNA (ceRNA) by sponging miR-222-5p, and the latter targeted at the 3′-untranslated region (UTR) of receptor-activity-modifying proteins 3 (RAMP3). Overall, this study identified a novel network of lncRNA LEF1-AS1/miR-222-5p/RAMP3 in SCI. Therefore, lncRNA LEF1-AS1/miR-222-5p/RAMP3 axis may be candidate target for SCI patients with significant clinical significance.

Presently, microglial cell apoptosis has been found to play a vital role in SCI development [22, 23]. Previous studies showed that microglial cells present in the spinal cord, are critically important in the pathogenesis of SCI [24]. TNF-α and IL-6 expression were found to be overexpressed in SCI tissues and aggravate damage of microglial cells [25]. Therefore, after in vivo and in vitro SCI model was established, their BBB scores were significantly downregulated than in sham-operated animals. Though lncRNA LEF1-AS1 was significantly upregulated in the SCI model, in this study it was observed that lncRNA LEF1-AS1 negatively impact on SCI progression. Also, recent assessments have revealed that LncRNA LEF1-AS1 located in chromosome 4q25 is upregulated in lung cancer hence promote lung cancer cell apoptosis by regulating PTEN expression [26]. Also, knockdown of lncRNA LEF1-AS1 inhibits the progression of squamous cell carcinoma progression via targeting the Hippo signaling pathway [27]. Consequently, the LEF-AS1 knockdown could partially restore BBB score in SCI rats. Furthermore, in vivo experiments suggested that knockdown of LEF-AS1 could decrease the apoptosis and inflammation response.

Recent studies have shown that lncRNAs interact with miRNAs to regulate gene expression as complementary endogenous RNAs (ceRNAs). Therefore, our study found that miR-222-5p was a target of lncRNA LEF-AS1 through the bioinformatics tool and luciferase activity experiments. However, knockdown of miR-222-5p could partially attenuate the inhibitory effects of si-LEF-AS1 in the anti-apoptosis of microglial cells. In general, our results suggested that lncRNA LEF-AS2 served as a ceRNA of miR-222-5p to inhibit downstream gene expression. According to Chen et al. [28], miR-222-5p has inhibitory effects on staphylococcal enterotoxin B-induced inflammatory response in lung injury. Also, Bai et al. [29] showed that miR-222 possesses a regulatory role in brain injury and inflammation due to intracerebral hemorrhage. Moreover, miR-222-5p was downregulated in the SCI model, thus, suggesting miR-222-5p having a positive role in inhibiting LPS-mediated microglia cells apoptosis.

According to our findings, RAMP3 was a target gene of miR-222-5p. However, a previous study had shown that RAMP3 could induce reactive oxygen species and apoptosis [30]. As verified by our results, RAMP3 was a functional target of miR-222-5p and negatively regulated by the latter. LEF-AS1 overexpression augmented the RAMP3 expression level. Therefore, it is hypothesized that LEF-AS1/miR-222-5p modulates SCI by modulating RAMP3.

Some limitations of this study are as follows: (1) we did not perform knock down or overexpression experiments for RAMP3 gene; (2) one mRNA may be regulated by several lncRNAs at the same time, and that a single lncRNA can also affect several mRNAs simultaneously; and (3) crucial downstream signaling pathway of RAMP3 was not perform and need for further research. Future studies should focus on the following downstream pathway of RAMP3 in regulating microglia cells apoptosis and inflammation.

In summary, using both in vivo and in vitro models, we revealed that lncRNA LEF1-AS1 was upregulated after SCI. Moreover, knockdown of LEF1-AS1 could alleviate LPS-induced microglia cells apoptosis and inflammation injury by regulating the miR-222-5p/RAMP3 axis. Taken together, these findings identified a novel network of LEF1-AS1/miR-2222-5p/RAMP3 in SCI, which has the potential to serve as a new therapeutic target for SCI.

## Data Availability

We state that the data will not be shared since all the raw data are present in the figures included in the article.

## Abbreviations

SCI: Spinal cord injury
LPS: Lipopolysaccharide
SD: Sprague-Dawley
ceRNA: Competitive endogenous RNA (ceRNA)
lncRNAs: Long non-coding RNAs
LEF1: Lymphoid enhancer-binding factor 1 (LEF1)
LEF1-AS1: Lymphoid enhancer-binding factor 1 (LEF1) antisense RNA 1
miRNAs: MicroRNAs
LINGO-1: Ig domain-containing NOGO receptor-interacting protein 1
UTR: Untranslated region
RAMP3: Receptor-activity-modifying proteins 3
FBS: Fetal bovine serum
shRNA: Short-hairpin RNA
CCK-8: Cell count kit-8
PVDF: Polyvinylidene fluoride
WT: Wild-type
MUT: Mutant
